# Selective Type 2 Respiratory Failure Followed by Ocular Myasthenia Gravis Diagnosed by Ice Pack Test: A Case Report

**DOI:** 10.7759/cureus.4927

**Published:** 2019-06-17

**Authors:** Hitanshu Dave, Rupak Desai, Sanggita Checker, Priyank J Yagnik

**Affiliations:** 1 Internal Medicine, Hackensack Meridian Health - Jersey Shore University Medical Center, Neptune City, USA; 2 Cardiology, Atlanta Veterans Affairs Medical Center, Decatur, USA; 3 Pulmonary Medicine and Critical Care, Wockhardt Hospital, Mumbai, IND; 4 Pediatrics, University of Kansas School of Medicine, Wichita, USA

**Keywords:** type 2 respiratory failure, myasthenic ptosis, ocular myasthenia, ice pack test, breathlessness, myasthenia gravis

## Abstract

Myasthenia gravis is an autoimmune neuromuscular disorder that can present with skeletal muscle involvement, ocular muscles involvement and can progress to respiratory muscle paralysis. Here, we present a unique case of type 2 respiratory failure due to myasthenia gravis with the delayed ocular presentation. A 46-year-old female patient presented to the outpatient clinic with complaints of dyspnea. On further evaluation, she was found to be hypercapnic on arterial blood gas analysis with no muscular weakness in any of the limbs. The patient further progressed to ocular symptoms. With the use of an ice pack test, a bedside test for improvement of ptosis, the patient was diagnosed with myasthenic ptosis preventing further progression of type 2 respiratory failure and intubation. With this case report, we emphasize the critical role of such simple bedside test in timely diagnosis and management of myasthenia gravis while awaiting the final results.

## Introduction

Myasthenia gravis can invariably present with complications as the presenting symptoms. Herein, we report a case where the patient presented with the symptoms of type 2 respiratory failure only and there was a need for rapid diagnosis and treatment as we expected a delay in serology reports. Thus, a bedside test, ice pack test, came as a savior and could reliably diagnose myasthenia gravis with a delayed ocular presentation [1]. Ice pack is a simple and non-invasive test used for ocular myasthenia, possible physiological mechanism behind the local cooling of the muscle is that it improves muscular contraction by decreasing the activity of acetylcholinesterase enzyme thus acetylcholine stays in the synaptic cleft for a longer time, as well as it improves the presynaptic transmission of acetylcholine by increasing Ca^2+^ in the nerve fiber hence it ameliorates excitation-contraction coupling of the muscles. There is a visible improvement in the ptosis which can be measured before and after the test. Given the multiple mechanisms of improving postsynaptic transmission, wide ranges of sensitivities (80-100%) and specificities (25-100%) are noted [2].

## Case presentation

A 46-year-old female with a known history of hypothyroidism for 10 years presented in the outpatient department with dry cough for one week, fever for two days and breathlessness for 10 days which has worsened over the last two days. She had no history of power loss in any of her limbs. She had no history of smoking. She had no family history of any autoimmune disorders.

She visited a primary care physician one day earlier and was recommended arterial blood gas evaluation to detect the degree of respiratory impairment and possible etiologies. The arterial blood gas analysis report showed a pH of 7.3, pCO_2_ of 60 mmHg, pO_2_ of 52 mmHg and the patient was advised admission considering a high level of pCO_2_. On admission, blood samples were drawn for complete blood count, renal function test, liver function test, and thyroid stimulating hormone; all of which showed normal results. To rule out the pulmonary thromboembolism, a cardiothoracic pulmonary angiogram was performed which showed posterobasal consolidation in both lower lobes with a thin rim of pleural effusion bilaterally and a few enlarged homogeneously enhancing lymph nodes in the perivascular and right paratracheal regions.

On admission, the patient was also started on non-invasive positive pressure ventilation, bronchodilators, and broad-spectrum antibiotics against pneumonia. Despite these interventions, the patient showed no improvement for the next 24 hours. The subsequent arterial blood gas analysis showed a pH of 7.2 with pCO_2_ of 105 mmHg. Breath holding counts decreased from 12 to 6, thus developing hypercapnia. In addition, the patient started developing drowsiness for which a neurology consultation was ordered to rule out other neurologic causes of type 2 respiratory failure. To rule out myasthenia gravis, various serological tests were performed including acetylcholine receptor antibodies, IgA antibodies, electromyography with the results expected to arrive the next day.

**Figure 1:**
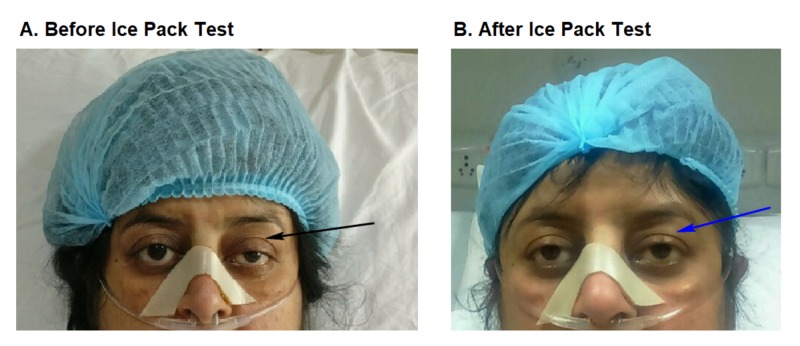
Ice Pack Test A: Black arrow is depicting ptosis before the ice pack test B: Blue arrow is showing resolved ptosis after the ice pack test. Note: Patient's photograph was taken after verbal consent of the patient.

Owing to the expected delay in the results, a bedside ice pack test was performed to diagnose myasthenia gravis. A positive response was elicited with the test (Figure 1) and neostigmine was started bearing in mind the deteriorating condition of the patient. Within 3 hours, pH was 7.3, pCO_2_ was 75 mmHg, pO_2_ was 70 mmHg with a gradual lowering of the pCO_2_. Her consciousness gradually improved and the non-invasive ventilator support was reduced within 6 hours. Next day all the results of the serological tests were obtained and turned out to be negative for myasthenia gravis. However, the electromyography test result was positive for two muscles confirming the diagnosis of myasthenia gravis. Detailed laboratory findings and investigation reports are shown in Table 1.

**Table 1:** Detailed laboratory and imaging workup

Laboratory/Imaging Test	Observed Findings (Values with Normal Range)
Hematology	
Total leukocyte count	8520 cells/cubic millimeter (4000–11000)
Hemoglobin	8.7 gram% (12-15)
Platelet count	359000 (150000–410000) /cubic millimeter
Neutrophils	63% (40–80)
Lymphocyte	32% (20–40)
Serology	
Human immunodeficiency virus immunoassay	Non-reactive
Hepatitis C virus immunoassay	Negative
Biochemistry	
Aspartate transaminase	21.45 units/liter (0–32)
Alanine transaminase	21.58 units/liter (0–35)
Total bilirubin	0.25 milligram/deciliter (0.3–1.2)
Total proteins (serum)	7.32 gram/deciliter (6.8–8.7)
Arterial Blood Gas	
pH	7.209 (7.35–7.44)
Pco_2_	105 mm Hg (34-45)
PO_2_	163 mm Hg (80 –108)
Serum Electrolytes	
Sodium	135 mill moles/liter (135–148)
Potassium	4.7 mill moles/liter (3.5–5.3)
Immunology	
Mi- 2 nuclear antigen	Negative
Sm/Ribo nucleotide protein	Negative
Scl–70 anti-topoisomerase 1 antibody	Negative
Anti- double stranded deoxy nucleotide acid	Negative
Histones	Negative
Ribosomes PO	Negative
Chest Radiography (anteroposterior sitting)	
Opacities and consolidation	Ill-defined opacity in left cost phrenic angle possible consolidation.
Lung fields	Clear and unremarkable
Computed Tomographic Pulmonary Angiogram	
Pulmonary thromboembolism	No evidence
Consolidation	Posterobasal consolidation in both lobes.
Echocardiography and Color Doppler	
Cardiac values	Normal
Heart chamber size	Normal
Left ventricular thickness	Normal
Pulmonary artery hypertension	Moderate
Antibody test	
Acetylcholine receptor antibodies	Not detected (0 – 0.25 nanomoles/liter)
Muscle-specific tyrosine kinase antibody	Not detected (<0.05 nanomoles/liter)

## Discussion

Myasthenia gravis is an autoimmune disorder that affects the skeletal muscles of the body by producing anti-acetylcholine esterase antibodies. These antibodies act on the receptors and prevent neuromuscular transmission [3]. Myasthenia gravis can present in various forms like generalized myasthenia, ocular myasthenia in which predominantly ocular muscles are involved or respiratory involvement which is the dreaded complication as it can lead to respiratory failure. This patient presented with respiratory myasthenia and progressed to ocular myasthenia gravis. Myasthenia gravis was one of the differential diagnoses for this case, but rapid diagnostic testing with anti-acetylcholinesterase antibodies would still take at least 24 hours to confirm or refute the diagnosis. Moreover, anti-acetylcholinesterase antibodies testing could also be a false negative. Thus, the ice pack test was employed for the diagnosis as it can be done at the bedside and doesn’t require much expertise or time. The principle of the test uses the cooling of the muscle thereby improving ptosis by inhibiting acetylcholinesterase enzyme [4]. Ice pack test can be accomplished in 2-5 minutes and ptosis should be evaluated at baseline and then checked after 5 minutes, if there is an improvement of > 2 millimeters then it is considered positive [5]. Literature has also reported the prior use of this test for myasthenic diplopia and ptosis in cases with ocular myasthenia gravis [6].

## Conclusions

In this case with a unique primary presentation of respiratory involvement, the ice pack test was used effectively to timely diagnose the delayed presentation of ocular myasthenia gravis with ptosis. This test can provide a quick bedside diagnosis and can be used rapidly in healthcare setups with the unavailability of modern-day tests or an expected delay in test results. The major drawback of the test is that it can be only used in patients with ocular myasthenia gravis. Effectiveness of the test is not significant in patients with skeletal muscle myasthenia gravis. If the test is conducted appropriately with the usage of proper technique and understanding of the interpretation of results, it very well can be effectual in the commencement of the early treatment. 
